# Prednisolone improves the response to primary endocrine treatment for advanced breast cancer.

**DOI:** 10.1038/bjc.1988.273

**Published:** 1988-11

**Authors:** R. D. Rubens, C. L. Tinson, R. E. Coleman, R. K. Knight, D. Tong, P. J. Winter, W. R. North

**Affiliations:** Imperial Cancer Research Fund, Clinical Oncology Unit, Guy's Hospital, London, UK.

## Abstract

Two hundred and twenty patients with progressive advanced breast cancer were given primary endocrine treatment (PET) according to menstrual status. Pre-menopausal patients received ovarian irradiation (O) and post-menopausal tamoxifen 10 mg bd (T). Patients were randomised to receive either no additional treatment or prednisolone 5 mg bd (P). Similar results were observed in each menstrual subgroup. In 194 evaluable patients, the response to PET + P was 49% and to PET alone 30% (P less than 0.01). P increased the median duration of response from 9 to 14 months (P less than 0.002) and the median time to disease progression from 5 to 9 months (P less than 0.001). Response to P after O or T alone occurred in only 2/62 (3%). Median survival in patients randomised to receive P at the outset of PET was prolonged by 4 months (P less than 0.05). The addition of P significantly improves the response to O or T in the treatment of advanced breast cancer.


*Br. J. Cancer* (1988), **58**, 626–630 © The Macmillan Press Ltd., 1988

### Prednisolone improves the response to primary endocrine treatment for advanced breast cancer

R.D. Rubens, C.L. Tinson, R.E. Coleman, R.K. Knight, D. Tong, P.J. Winter & W.R.S. North

*Imperial Cancer Research Fund, Clinical Oncology Unit, Guy's Hospital, London, SE1 9RT, UK.*

**Summary** Two hundred and twenty patients with progressive advanced breast cancer were given primary endocrine treatment (PET) according to menstrual status. Pre-menopausal patients received ovarian irradiation (O) and post-menopausal tamoxifen 10 mg bd (T). Patients were randomised to receive either no additional treatment or prednisolone 5 mg bd (P). Similar results were observed in each menstrual subgroup. In 194 evaluable patients, the response to PET+P was 49% and to PET alone 30% ($P<0.01$). P increased the median duration of response from 9 to 14 months ($P<0.002$) and the median time to disease progression from 5 to 9 months ($P<0.001$). Response to P after O or T alone occurred in only 2/62 (3%). Median survival in patients randomised to receive P at the outset of PET was prolonged by 4 months ($P<0.05$). The addition of P significantly improves the response to O or T in the treatment of advanced breast cancer.

For some years the preferred primary endocrine treatment for metastatic breast cancer has been ovarian ablation for pre-menopausal, and tamoxifen for post-menopausal, patients. Corticosteroids also have activity against breast cancer (Minton *et al.*, 1981) but the mechanism of action is not known for certain. It may be mediated by suppression of adrenal sex hormones and also have a direct action on cancer cells. When prednisolone was added to adjuvant ovarian irradiation after mastectomy for early breast cancer, it led to a significant reduction in recurrence rate and improved survival (Meakin *et al.*, 1979).

These observations led to a trial in this Unit to assess the contribution of prednisolone to the primary endocrine treatment of advanced breast cancer (Stewart *et al.*, 1982). Pre-menopausal patients with metastatic breast cancer were randomised to receive ovarian irradiation either alone or in combination with prednisolone, while post-menopausal patients received tamoxifen either alone or with prednisolone. The corticosteroid significantly improved the response frequency to primary endocrine treatment and, in post-menopausal patients, survival was prolonged. It was unclear from this trial whether using prednisolone sequentially, after tamoxifen alone, could have achieved similar results. A further observation was that prednisolone appeared to prevent the occurrence of hypercalcaemia and tumour 'flare' sometimes seen with tamoxifen alone. In that trial, the response to tamoxifen alone was unexpectedly low at 17% and the conclusion that prednisolone significantly improved the response frequency was only tentative. Accordingly, another trial was established to study further the contribution of prednisolone to primary endocrine treatment. This trial, in which the addition of prednisolone after failure of primary endocrine treatment alone is also studied, is reported here.

### Patients and methods

### *Patients*

Eligible patients had progressively locally recurrent and/or metastatic breast cancer, confirmed histologically and not controllable by local treatment. The disease was evaluable according to UICC criteria (Hayward *et al.*, 1977). Patients had had prior primary treatment by either mastectomy or tumour excision with radiotherapy for operable disease, or radiotherapy alone for primary locally advanced inoperable disease. Patients who had had adjuvant endocrine treatment were excluded. Patients might have had radiotherapy to metastatic sites, but none had had previous endocrine treatment or chemotherapy for recurrent or metastatic disease.

Steroid receptor information was available for most tumours. Oestrogen receptor (ER) and progesterone receptor (PgR) status was deemed positive for values of 5 or more fmol receptor $mg^{-1}$ cystosol protein by the method of King *et al.* (1979). Patients with tumours positive for one (ER+PgR− or ER−PgR+) or both (ER+PgR+) receptors or of unknown receptor status were eligible for the trial; patients with tumours known to be negative for both receptors were excluded.

Patients were ineligible for the trial if they were receiving systemic corticosteroid therapy or if they had done so in the previous year. They were excluded if there were medical contra-indications to the use of corticosteroids (e.g., diabetes mellitus, peptic ulcer) or if there had been previous malignancy other than non-melanomatous skin cancer or adequately treated carcinoma *in situ* of the cervix uteri. Potentially eligible patients with concurrent hypercalcaemia were only eligible if this could be controlled without the use of corticosteroids. Irrespective of other features of eligibility, patients in whom immediate chemotherapy was thought advisable because of rapidly progressing life-threatening disease (e.g., pulmonary infiltration or liver metastases with marked elevation of liver enzymes) were excluded.

### *Menstrual status*

Before randomisation, patients were stratified according to menstrual status in order to determine the appropriate endocrine therapy.

(a) Patients who had had a menstrual period within the previous 6 months were regarded as pre-menopausal, while if the last menstrual period was 6 months ago or longer, patients were deemed post-menopausal.
(b) Patients with a previous hysterectomy and retention of one or both ovaries were regarded as pre-menopausal if less then 50 years of age and post-menopausal if 50 years or over.
(c) Pre-menopausal patients who ceased menstruating whilst on adjuvant chemotherapy and then relapsed whilst receiving, or within 6 months of stopping, chemotherapy were considered pre-menopausal. If relapse occurred 6 months or more after stopping adjuvant chemotherapy, the menstrual status was assessed as in (a) above.

Correspondence: R.D. Rubens.
Received 2 February, 1988; and in revised form, 20 June 1988.

*Treatment*

Pre-menopausal patients were randomised to receive either (1) ovarian irradiation (1,200–1,500 cGy central dose in 4–5 fractions in 4–7 days by opposed fields to the whole pelvis) with prednisolone 5 mg bd starting on the first day of ovarian irradiation and continued until evidence of progressive disease (O+P); or (2) ovarian irradiation alone (O). On disease progression, patients receiving O alone were prescribed prednisolone 5 mg bd until there was further evidence of progressive disease. However, if on progression after O alone, rapidly progressive life-threatening disease made immediate chemotherapy advisable, this was instituted together with corticosteroids.

Post-menopausal patients were randomised to receive either (1) tamoxifen 10 mg bd + prednisolone 5 mg bd (T+P), continued until evidence of progressive disease; or (2) tamoxifen 10 mg bd alone (T), when, on progression of disease, T was continued and prednisolone 5 mg bd added until there was evidence of further disease progression. If on progression after T alone, rapidly progressive life-threatening disease made immediate chemotherapy advisable, this treatment was instituted together with corticosteroids.

As far as possible, treatment on relapse after prednisolone was standardised. Provided chemotherapy was not considered necessary, patients were prescribed aminoglutethimide 250 mg bd, rising to 500 mg bd after 4 weeks, together with hydrocortisone 20 mg bd as secondary endocrine treatment. Subsequent chemotherapy was according to current Unit protocols and, in general, the order of priority was Adriamycin alone, a combination of cyclophosphamide and methotrexate and 5-fluorouracil (CMF) or a combination of mitomycin C and vinblastine.

*Study parameters*

Before entry into the trial, a full physical examination was performed, measurements were made of all palpable lesions and visible lesions were photographed. All patients had a chest radiograph, isotope bone scan with radiographs of regions of increased uptake, a full blood count and a biochemical screen. Liver scans were only done if there was a suspicion of hepatic disease on clinical or biochemical grounds. Baseline lesions were selected for serial assessment. Patients were followed up at 4 weekly intervals with repeat assessment of baseline lesions and, when appropriate, photographs at each visit. Haematological and biochemical screens, chest radiographs and bone scans were repeated at every 3 months.

*Response criteria*

Objective response was assessed by UICC criteria (Hayward *et al.*, 1977). Duration of response was dated from the start of treatment until either new lesions appeared or any one existing lesion increased by 25% or more above the smallest size recorded or there was a definite deterioration in evaluable, but non-measurable, lesions (photographs or radiographs). These dates were also used for all evaluable patients to compute curves for time to disease progression. Survival was from the date of start of treatment to death. Survival and duration of response were analysed by the log-rank test. The significance of differences between binary variables was calculated by the chi-squared test for contingency tables. The records of patients on this trial were reviewed by an external assessor.

### Results

From 30 November 1981 to 28 August 1986, 220 patients entered this study. One hundred and ninety-four patients are evaluable after 26 had been excluded for the following reasons: at review, 6 patients were found to have been ineligible, 2 were lost to follow-up, 7 had had confounding treatment (6 corticosteroids, 1 interferon), and 11 had inadequate information for assessment of response (these patients are included in the analysis of survival). The minimum follow-up on any patient was 14 months.

The characteristics of the evaluable patients are shown in Table I, previous treatments in Table II and sites of disease at the start of treatment in Table III. These features are well balanced for the treatment groups according to whether prednisolone was combined with primary endocrine treat-

**Table I** Patient characteristics

| | Treatment group | | | |
|---|---|---|---|---|
| | *Pre-menopausal* | | *Post-menopausal* | |
| | *O+P* | *O→P* | *T+P* | *T→P* |
| Total patients assessable | 16 | 15 | 85 | 78 |
| Mean age at entry (years) | 40 | 43 | 63 | 62 |
| Stage at presentation (no. pts) | | | | |
| I & II (operable) | 14 | 13 | 67 | 57 |
| III & IV (advanced) | 2 | 2 | 18 | 21 |
| Post-operative disease-free interval | | | | |
| Median (months) | 21 | 32 | 20 | 21 |
| Range | 0–86 | 0–92 | 0–198 | 0–248 |
| Time from initial diagnosis to entry | | | | |
| Median (months) | 34 | 35 | 36 | 35 |
| Range | 0–100 | 0–152 | 0–203 | 0–248 |
| Steroid receptor status[a] (no. pts) | | | | |
| ER+PgR+ | 10 | 8 | 43 | 41 |
| ER+PgR− | 3 | 2 | 17 | 20 |
| ER−PgR+ | 1 | 0 | 2 | 2 |
| Unknown | 2 | 5 | 23 | 15 |

[a]When this information was available on both primary and metastatic tumour, that on the latter has been used in the analysis.

**Table II** Previous treatment

| | Treatment group | | | |
|---|---|---|---|---|
| | *Pre-menopausal* | | *Post-menopausal* | |
| | *O+P* | *O→P* | *T+P* | *T→P* |
| Modified radical mastectomy | 12 | 10 | 49 | 51 |
| Excision and radiotherapy | 1 | 3 | 19 | 6 |
| Adjuvant chemotherapy | 2 | 0 | 17 | 10 |
| Mephalan | 0 | 0 | 6 | 3 |
| CMF | 2 | 0 | 11 | 7 |
| Primary radiotherapy | 2 | 1 | 8 | 5 |
| Alone | 2 | 1 | 6 | 3 |
| With chemotherapy | 0 | 0 | 2 | 2 |
| Radiotherapy to metastases | 6 | 8 | 23 | 26 |

**Table III** Sites of disease at start of treatment

| | Treatment group | | | |
|---|---|---|---|---|
| | *Pre-menopausal* | | *Post-menopausal* | |
| | *O+P* | *O→P* | *T+P* | *T→P* |
| Skin | 7 | 8 | 33 | 26 |
| Breast | 4 | 4 | 21 | 21 |
| Lymphatic | 4 | 7 | 35 | 32 |
| Bone | 6 | 10 | 52 | 49 |
| Lung | 3 | 3 | 22 | 17 |
| Pleura | 1 | 3 | 21 | 13 |
| Liver | 0 | 0 | 7 | 9 |
| Other | 0 | 1 | 2 | 4 |

ment (O+P and T+P) or given sequentially on disease progression (O→P and T→P).

The numbers of patients in each of the response categories for primary endocrine treatment (O or T) with or without prednisolone (P) is shown in Table IV. In premenopausal patients, the response to O is increased by the addition of P (27% *vs.* 63%; $0.02<P<0.05$). Moreover, the median duration of response is increased from 9 to 20 months ($P=0.02$) and the median time to disease progression from 4 to 14 months ($P=0.006$). In post-menopausal patients, similar trends were observed. Response frequency to T alone was 31% and to T+P 46% ($0.05<P<0.1$) while prednisolone significantly increased both the median duration of response (10 *vs.* 14 months; $P=0.02$) and the median time to disease progression (4 *vs.* 8 months, $P=0.02$).

Because of the identical clinical objectives of primary endocrine treatment and the similarity of effects in pre- and post-menopausal patients, the above results have been combined. Primary endocrine treatment with prednisolone (O+P and T+P) gives a response frequency of 49/101 (49%), while without prenidosolone (O and T) it is 28/93 (30%) ($P<0.01$). The median duration of response in responding patients was 9 months for primary endocrine therapy alone compared to 14 months when combined with prednisolone ($P<0.002$; Figure 1) and the median times to disease progression for patients in these groups were 5 and 9 months respectively ($P<0.001$; Figure 2).

The objective regressions at each site of assessable disease are shown in Table V and there is general tendency for the responses to be higher in the presence of prednisolone at the various sites. Response in relation to steroid receptor status is summarised in Table VI which shows a high overall response rate for ER+PgR+tumours (46%) and those of unknown status (44%), but a low rate for ER+PgR− tumours (19%).

Seventy-two patients who have progressed after primary endocrine treatment alone have had prednisolone 5 mg bd prescribed. Sixty-two are assessable of whom only 2 (3%) achieved an objective regression (both partial responses lasting 15+ and 22 months). One was pre- and the other post-menopausal; both had responded to primary endocrine treatment for 17 and 11 months respectively. Thirty-seven patients have received aminoglutethimide and hydrocortisone as secondary endocrine treatment and 7 (19%) achieved an objective response; 6 had responded to primary endocrine treatment.

The median survival of patients randomised to receive prednisolone at the outset of primary endocrine treatment was significantly increased from 17 to 21 months ($P<0.05$; Figure 3). This effect was statistically significant in pre-menopausal patients (17 *vs.* 66 months; $P=0.04$), but not in post-menopausal (17.5 *vs.* 21 months, $P=0.3$).

Primary endocrine treatment either with or without prednisolone was very well tolerated and any side-effects were mild. Those side-effects probably attributable to treatment are summarised in Table VII. Weight gain only gave rise to complaints when prednisolone was prescribed and affected 15% of such patients. Interestingly, hot flushes were more common in the presence of prednisolone suggesting that this agent enhances the anti-oestrogenic effect of primary endocrine treatment. The incidence of tumour 'flare' and hypercalcaemia due to tamoxifen was the same whether or not prednisolone was given. Prednisolone was added in 3 patients who developed hypercalcaemia on tamoxifen alone.

**Figure 1** Duration of response to primary endocrine treatment (——— with prednisolone; - - - without prednisolone; $P<0.002$).

**Figure 2** Time to progression of disease after start of primary endocrine treatment (——— with prednisolone; - - - - - without prednisolone; $P<0.001$).

**Table V** Objective regressions at each site of assessable disease[a]

| | *Treatment group* | | | |
|---|---|---|---|---|
| | *Pre-menopausal* | | *Post-menopausal* | |
| | *O+P* | *O* | *T+P* | *T* |
| Skin | 5/7 | 2/8 | 13/33 | 8/26 |
| Breast | 3/3 | 1/4 | 11/22 | 12/21 |
| Lymphatic | 2/4 | 4/7 | 20/35 | 17/30 |
| Bone | 4/6 | 0/8 | 12/41 | 6/41 |
| Lung | 1/3 | 0/3 | 10/21 | 3/16 |
| Pleura | 1/1 | 0/1 | 4/15 | 2/6 |
| Liver | 0/0 | 0/0 | 1/4 | 1/9 |
| Other | 0/0 | 0/1 | 1/2 | 0/0 |

[a]Numerator = number of patients with objective regressions at stated site; denominator = number of patients with assessable disease at stated site at start of treatment.

**Table IV** Numbers of patients in the response categories for primary endocrine treatment

| | *Treatment group* | | | | | | | |
|---|---|---|---|---|---|---|---|---|
| | *Pre-menopausal* | | | | *Post-menopausal* | | | |
| | *O+P* | | *O* | | *T+P* | | *T* | |
| Complete response | 2 | 10 (63%) | 1 | 4 (27%) | 5 | 39 (46%) | 3 | 24 (31%) |
| Partial response | 8 | | 3 | | 34 | | 21 | |
| No change | 3 | | 5 | | 22 | | 25 | |
| Progressive disease | 3 | | 6 | | 24 | | 28 | |

**Table VI** Objective regressions in relation to steroid receptor status

| | Treatment group | | | |
|---|---|---|---|---|
| | *Pre-menopausal* | | *Post-menopausal* | |
| *Receptor phenotype* | *O+P* | *O* | *T+P* | *T* |
| ER+PgR+ | 7/10 | 3/8 | 20/43 | 17/41 |
| ER+PgR− | 1/3 | 0/2 | 5/17 | 2/20 |
| ER−PgR+ | 1/1 | 0/0 | 1/2 | 0/2 |
| Unknown | 1/2 | 1/5 | 13/23 | 5/15 |

**Figure 3** Survival from start of primary endocrine treatment (—— with prednisolone; ----- without prednisolone; $P<0.05$).

**Table VII** Side-effects of treatment

| | Treatment group | | | |
|---|---|---|---|---|
| | *Pre-menopausal* | | *Post-menopausal* | |
| | *O+P* | *O* | *T+P* | *T* |
| Weight gain | 6 | 0 | 10 | 0 |
| Hot flushes | 7 | 1 | 6 | 2 |
| Tumour flare | 0 | 0 | 1 | 1 |
| Headaches | 1 | 1 | 1 | 0 |
| Fluid retention | 0 | 0 | 5 | 0 |
| Hypercalcaemia | 0 | 0 | 4 | 4 |
| Gastro-intestinal | 1 | 2 | 4 | 2 |
| [a]Other | 0 | 0 | 5 | 1 |

[a]Nocturia, dry mouth, bruising, night cramps.

No patient had to have treatment interrupted or discontinued because of side-effects.

### Discussion

The rationale for, and the intention of, endocrine treatment for breast cancer has been to reduce the oestrogen environment of the tumour. This can be achieved in various ways including removing the source of hormones (ovarian ablation) or blocking their receptors (tamoxifen). It is now apparent that the mechanisms of action of tamoxifen are more complex than originally thought. A cytostatic effect has been observed in the complete absence of oestrogen, suggesting the possibility of receptor mediated anti-growth factor activity (Vignon *et al.*, 1987). Tamoxifen has also been shown to induce breast cancer cells to secrete transforming growth factor-beta which inhibits cell growth (Knabbe *et al.*, 1987). Such mechanisms could underly the observed efficacy of tamoxifen as adjuvant treatment in oestrogen receptor negative tumours (Wilson *et al.*, 1985). Nevertheless, in the palliation of advanced breast cancer, response to tamoxifen is uncommon with receptor negative tumours (Stewart *et al.*, 1982) and rare in non-hormone-dependent cancers (Jackson & Lowery, 1987). Hence the prime objective of endocrine treatment for advanced breast cancer still seems to be a lowering of the tumour's oestrogen status which currently is best achieved by ovarian ablation in pre-menopausal and tamoxifen in post-menopausal patients. These treatments give similar results and combining these menstrual subsets as primary endocrine treatment in this trial was considered valid.

The results reaffirm those of the previous trial (Stewart *et al.*, 1982) and show that the addition of prednisolone to primary endocrine treatment significantly increases the response frequency from an expected 30% to 49%. The duration of response and survival are also increased and particularly significant in pre-menopausal patients. The response to prednisolone after failure of primary endocrine treatment alone was poor. Response to aminoglutethimide as secondary endocrine treatment was lower than expected, but the gain achieved by combining prednisolone with initial endocrine therapy is not outweighed as shown by the survival data. Unlike the previous trial, prednisolone did not prevent hypercalcaemia or tumour 'flare'.

The mechanism of action of prednisolone is uncertain, but the larger number of patients experiencing hot flushes when this agent was used suggests that it acts, at least in part, by enhancing the anti-oestrogenic effect of treatment. In the previous trial, it was shown that prednisolone had a marked, and expected, inhibitory effect on serum dehydroepiandrosterone sulphate levels and it also marginally reduced the levels of serum oestradiol in post-menopausal women receiving tamoxifen (Blackburn *et al.*, 1984). Prednisolone had no effect on the serum levels of luteinising hormone, follicular stimulating hormone or prolactin. Because of the recent interest in the role of free-oestradiol in the aetiology and clinical course of breast cancer and its availability reflected in serum levels of sex hormone binding globulin (SHBG), SHGB levels in the stored sera of patients in the previous trial have been studied (Wang *et al.*, 1988). In pre-menopausal patients, prednisolone had no effect on the reduction in SHGB levels seen after ovarian ablation. In post-menopausal patients, tamoxifen led to a rapid rise in SHBG levels, but this was markedly dampened by prednisolone. Hence, although treatment by ovarian ablation and tamoxifen is designed to reduce oestrogen status in women with metastatic breast cancer, the action of prednisolone on SHBG would not appear to contribute to this effect.

Two clinical trials have now shown that prednisolone improves the response to primary endocrine treatment and this is achieved without significant side effects. The mechanism of action is not known, but is probably complex involving effects on both oestrogen status and directly on the cancer cell. The optimal dose of prednisolone is not known, but higher doses than used here would probably lead to an unacceptable incidence of adverse effects. We now routinely prescribe prednisolone with ovarian ablation in pre-menopausal, and tamoxifen in post-menopausal, patients for the treatment of metastatic breast cancer, provided that there are no contra-indications to corticosteroids.

We are grateful to Dr R. J. B. King for the steroid receptor analyses, Professor N. Gad-el-Mawla for reviewing the clinical records and Miss Z. Doran for help with data retrieval.

### References

BLACKBURN, A.M., WANG, D.Y., BULBROOK, R.D. & 4 others (1984). Effect of prednisolone on hormone profiles during primary endocrine treatment of advanced breast cancer. *Cancer Treatment Rep.*, **68**, 1447.

HAYWARD, J.L., RUBENS, R.D., CARBONE, P.P., HEUSON, J.-C., KUMAOKA, S. & SEGALOFF, A. (1977). Assessment of response to therapy in advanced breast cancer. *Br. J. Cancer*, **35**, 292.

JACKSON, I.M. & LOWRY, C. (1987). Clinical use of anti-oestrogens. In *Pharmacology and Clinical Uses of Inhibitors of Hormone Secretion and Action*, Furr, B.J.A. & Wakeling, A.E. (eds) p. 87. Bailliere Tindall: London.

KING, R.G.B., REDGRAVE, R., HAYWARD, J.L., MILLIS, R.R. & RUBENS, R.D. (1979). The measurement of receptors for oestradiol and progesterone in human breast cancers. In *Steroid Receptor Assays in Breast Tumours; Methodological and Clinical Aspects*, King, R.J.B. (ed) p. 55. Alpha Omega: Cardiff.

KNABBE, C., LIPPMAN, M.E., WAKEFIELD, L.M. & 4 others (1987). Evidence that transforming growth factor-beta is a hormonally regulated negative growth factor in human breast cancer cells. *Cell*, **48**, 417.

MEAKIN, J.W., ALLT, W.E.C., BEALKE, F.A. & 11 others (1979). Ovarian irradiation and prednisolone therapy following surgery and radiotherapy for carcinoma of the breast. *Can. Med. Assoc. J.* **120**, 1221.

MINTON, M.J., KNIGHT, R.K., RUBENS, R.D. & HAYWARD, J.L. (1981). Corticosteroids for elderly patients with breast cancer. *Cancer*, **48**, 883.

STEWART, J., KING, R., HAYWARD, J. & RUBENS, R. (1982). Estrogen and progesterone receptors: correlation of response rates, site and timing of receptor analysis. *Breast Cancer Res. Treat.*, **2**, 243.

STEWART, J.F., RUBENS, R.D., KING, R.J.B. & 6 others (1982). Contribution of prednisolone to the primary endocrine treatment of advanced breast cancer. *Eur. J. Cancer Clin. Oncol.*, **18**, 1307.

VIGNON, F., BOUTON, M.M. & ROCHEFORT, H. (1987). Antiestrogens inhibit the mitogenic effect of growth factors on breast cancer cells in the total absence of estrogens. *Biochim. Biophys. Res. Comm.*, **146**, 1502.

WANG, D.Y., RUBENS, R.D., CLARK, G.M.G., MOORE, J.W. & BULBROOK, R.D. (1988). Effects of prednisolone on sex hormone binding globulin during primary endocrine treatment of advanced breast cancer. *Breast Cancer Res. Treat.*, **11**, 67.

WILSON, A.J., BAUM, M., BRINKLEY, D.M. & 8 others (1985). Six-year results of a controlled trial of tamoxifen as single adjuvant agent in management of early breast cancer. *World J. Surg.*, **9**, 756.


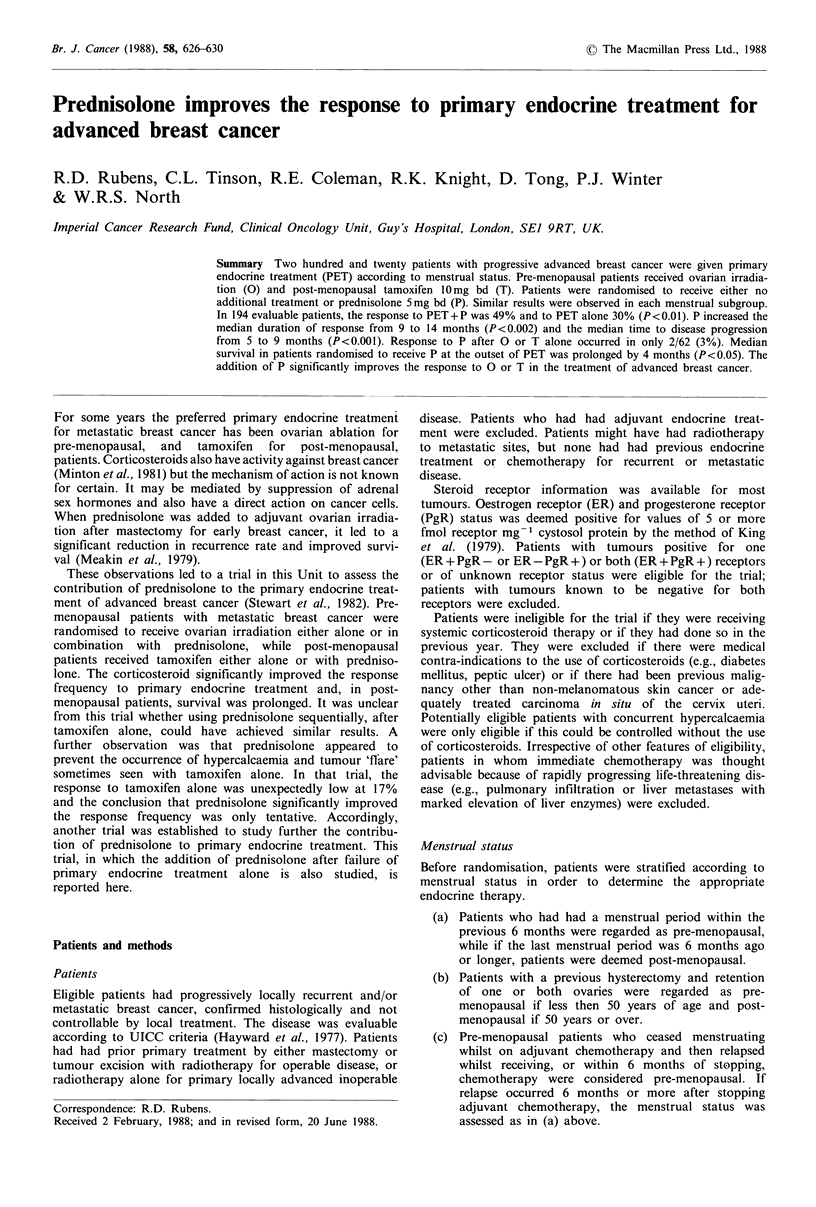

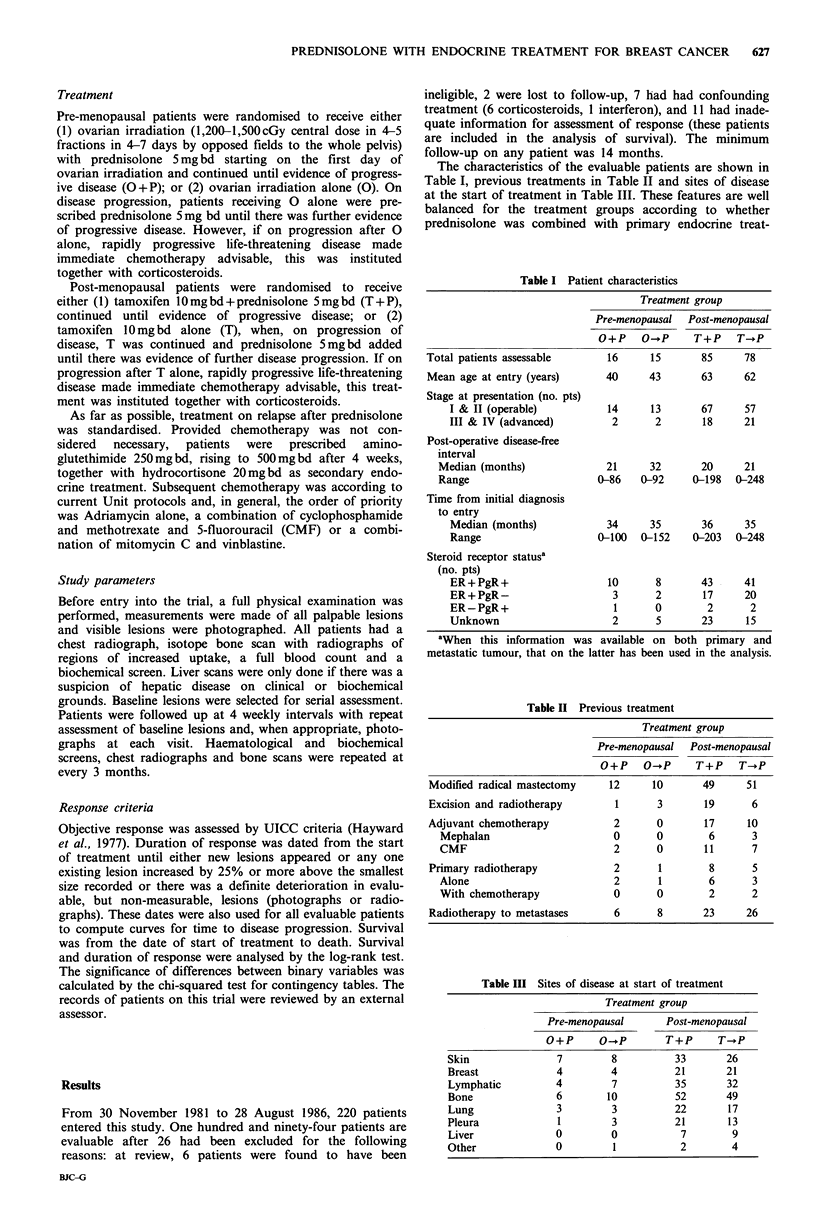

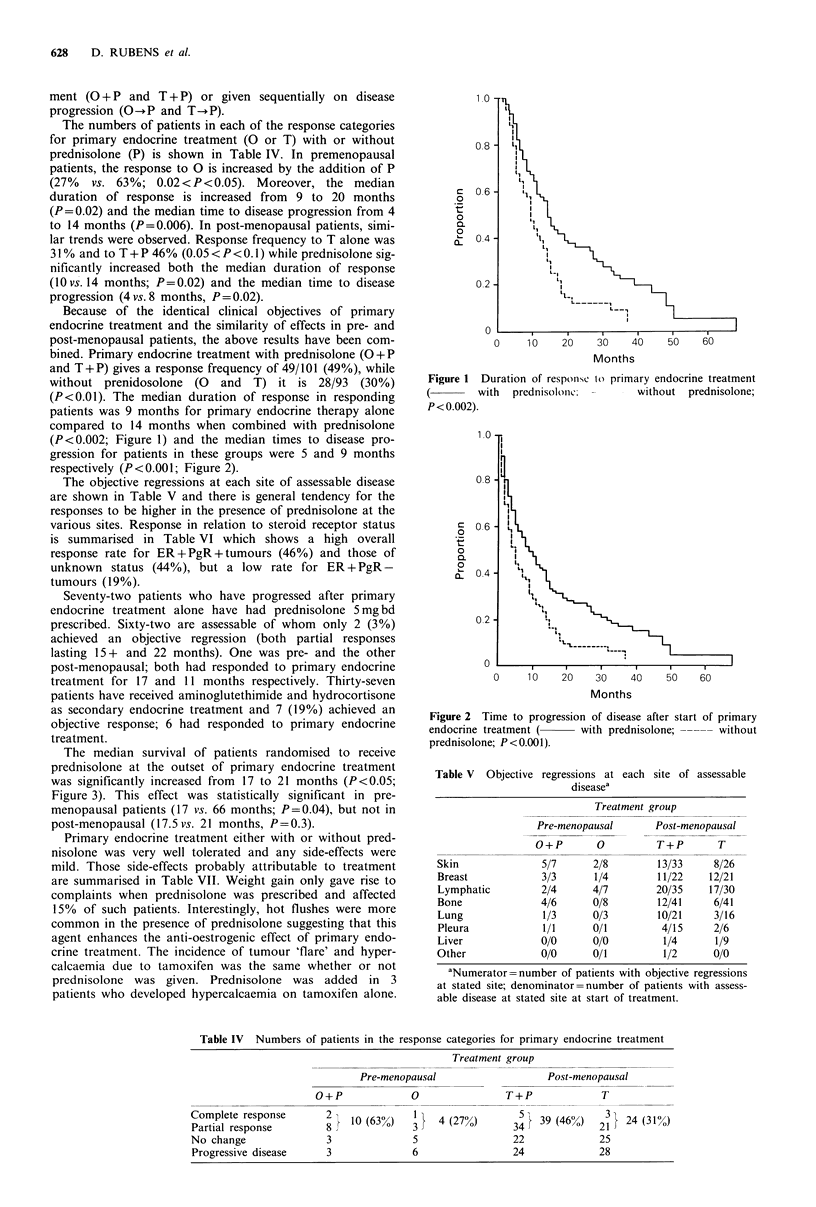

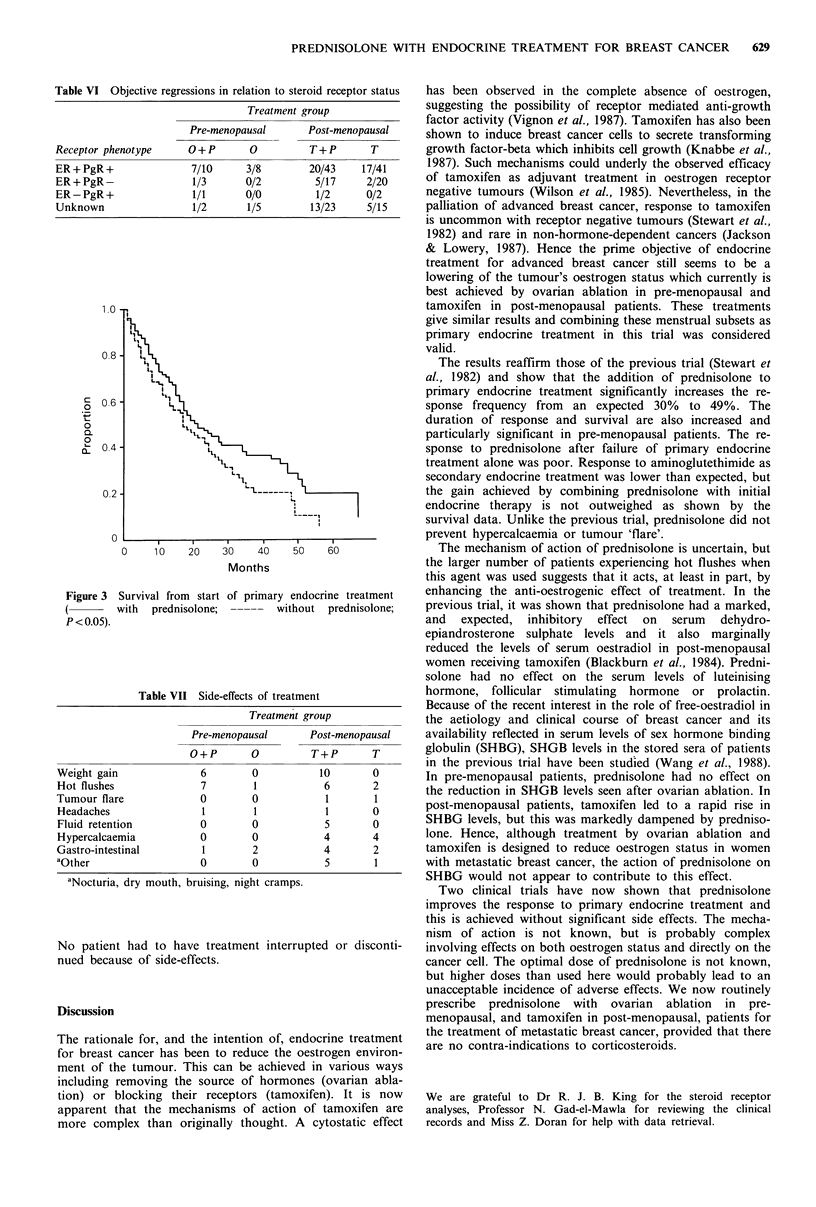

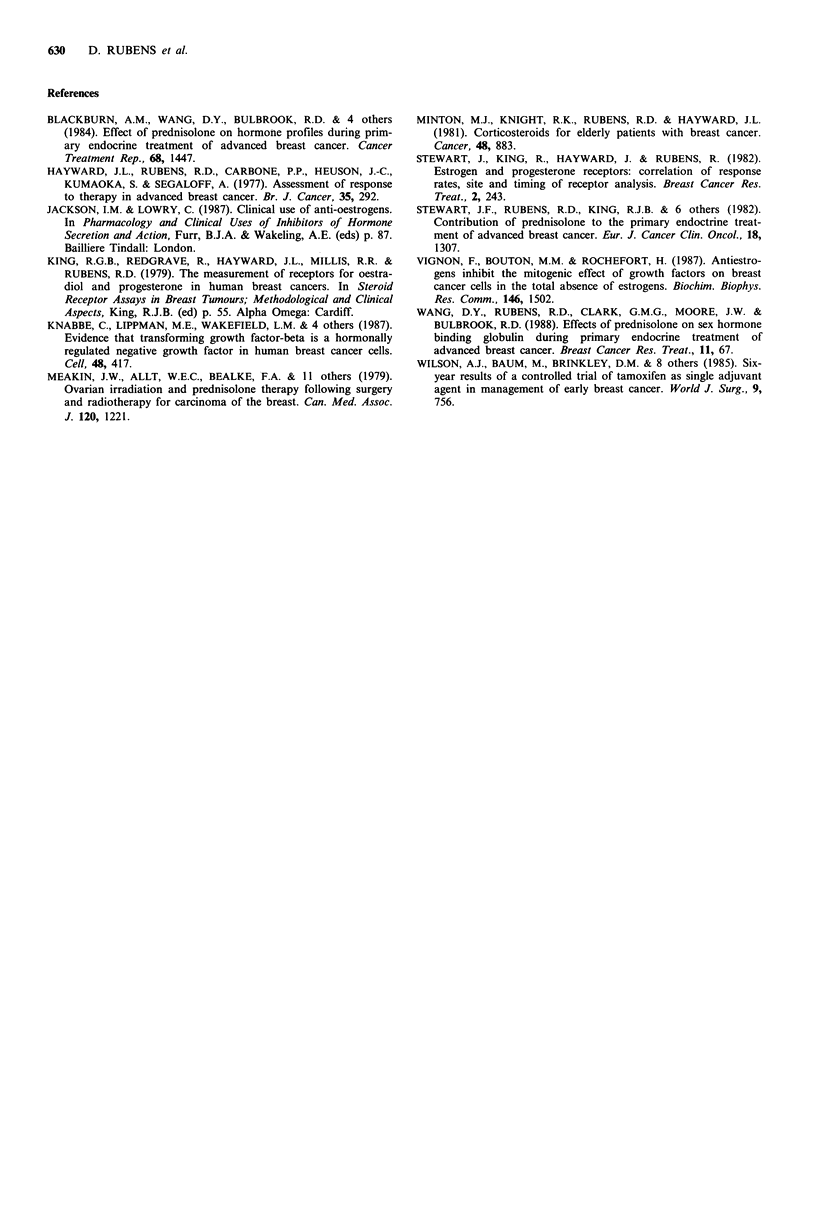


## References

[OCR_00601] Blackburn A. M., Wang D. Y., Bulbrook R. D., Thomas B. S., Kwa H. G., Hoare S. A., Rubens R. D. (1984). Effect of prednisolone on hormone profiles during primary endocrine treatment of advanced breast cancer.. Cancer Treat Rep.

[OCR_00607] Hayward J. L., Carbone P. P., Heusen J. C., Kumaoka S., Segaloff A., Rubens R. D. (1977). Assessment of response to therapy in advanced breast cancer.. Br J Cancer.

[OCR_00625] Knabbe C., Lippman M. E., Wakefield L. M., Flanders K. C., Kasid A., Derynck R., Dickson R. B. (1987). Evidence that transforming growth factor-beta is a hormonally regulated negative growth factor in human breast cancer cells.. Cell.

[OCR_00631] Meakin J. W., Allt W. E., Beale F. A., Brown T. C., Bush R. S., Clark R. M., Fitzpatrick P. J., Hawkins N. V., Jenkin R. D., Pringle J. F. (1979). Ovarian irradiation and prednisone therapy following surgery and radiotherapy for carcinoma of the breast.. Can Med Assoc J.

[OCR_00637] Minton M. J., Knight R. K., Rubens R. D., Hayward J. L. (1981). Corticosteroids for elderly patients with breast cancer.. Cancer.

[OCR_00648] Stewart J. F., Rubens R. D., King R. J., Minton M. J., Steiner R., Tong D., Winter P. J., Knight R. K., Hayward J. L. (1982). Contribution of prednisolone to the primary endocrine treatment of advanced breast cancer.. Eur J Cancer Clin Oncol.

[OCR_00642] Stewart J., King R., Hayward J., Rubens R. (1982). Estrogen and progesterone receptors: correlation of response rates, site and timing of receptor analysis.. Breast Cancer Res Treat.

[OCR_00654] Vignon F., Bouton M. M., Rochefort H. (1987). Antiestrogens inhibit the mitogenic effect of growth factors on breast cancer cells in the total absence of estrogens.. Biochem Biophys Res Commun.

[OCR_00660] Wang D. Y., Rubens R. D., Clark G. M., Moore J. W., Bulbrook R. D. (1988). Effects of prednisolone on sex hormone binding globulin during primary endocrine treatment of advanced breast cancer.. Breast Cancer Res Treat.

[OCR_00666] Wilson A. J., Baum M., Brinkley D. M., Dossett J. A., McPherson K., Patterson J. S., Rubens R. D., Smiddy F. G., Stoll B. A., Richards D. (1985). Six-year results of a controlled trial of tamoxifen as single adjuvant agent in management of early breast cancer.. World J Surg.

